# Artificial Intelligence‐Driven Hemodynamic Monitoring of Simulated Bruxism Using Functional Near‐Infrared Spectroscopy: A Preliminary Study

**DOI:** 10.1111/cns.70619

**Published:** 2025-09-22

**Authors:** Noor Fatima, Zia Mohy Ud Din, Abdullah Al Aishan, Jahan Zeb Gul

**Affiliations:** ^1^ Department of Biomedical Engineering Air University Islamabad Pakistan; ^2^ Department of Basic Medical Sciences, College of Applied Sciences King Khalid University Abha Saudi Arabia; ^3^ Department of Electronic Engineering Maynooth University Maynooth Ireland

**Keywords:** bruxism, fNIRS, hemodynamic assessment, machine learning

## Abstract

**Background:**

Sleep‐related and neuromuscular conditions affect the daily lives of individuals as they impact physical and cognitive well‐being. While not classified as a disorder, bruxism has emerged as a prevalent condition characterized by involuntary teeth grinding and jaw clenching, occurring either during sleep or wakefulness. Often left unnoticed, this unconscious behavior can contribute to severe dental damage, facial muscle fatigue, and temporomandibular joint disorders. These consequences require early detection and intervention to prevent long‐term complications. Traditionally, polysomnography (PSG) is widely used for bruxism assessments as it gives insights into the multimodal physiological data, but it lacks direct spatial mapping of neural regions involved in rhythmic masticatory muscular activity (RMMA) associated with bruxism.

**Methodology:**

This research introduces functional Near Infrared Spectroscopy (fNIRS) as a neuroimaging tool to monitor cortical activity associated with RMMA, distinguishing bruxism from other masticatory activities. The data were acquired in a controlled simulated paradigm setup from 10 subjects in three trials via a 20‐channeled optode setup of fNIRS placed over the motor cortex region. A total of 12 temporal and frequency domain features were optimized by employing techniques of feature selection, feature importance, and feature reduction. Furthermore, synthetic data augmentation techniques of Synthetic Minority Oversampling Technique (SMOTE), Synthetic Minority Oversampling Technique for Nominal features (SMOTEN), and Adaptive Synthetic sampling (ADASYN) were compared to five machine learning classifiers including k‐Nearest Neighbors (kNN), Logistic Regression (LR), Naive Bayes (NB), Decision Tree (DT), and Random Forest (RF).

**Results:**

The kNN outperformed in detecting simulated bruxism among other mandible joint movements with an accuracy of 92%.

**Conclusion:**

The findings highlight the potential of fNIRS as a tool for identifying and distinguishing bruxism‐like motor activities from other jaw movements, contributing to the timely management and detection of bruxism in future studies.

## Graphical abstract

This research explores functional near‐infrared spectroscopy (fNIRS) as a novel technique to monitor bruxism. The fNIRS data were recorded over the motor cortex to record different masticatory activities and monitor their corresponding hemodynamic states. Lastly, extracted features were optimized and oversampled to detect bruxism using machine learning classifiers.

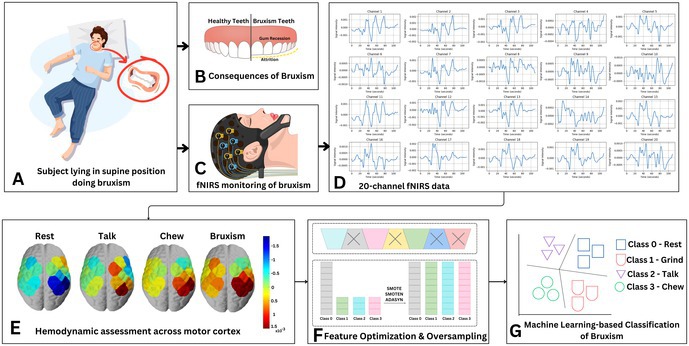

## 1 Introduction

Around one in five individuals globally experience bruxism, according to a 2024 meta‐analysis, which estimates the global prevalence of bruxism (sleep and awake) at 22.22%, with the prevalence of sleep bruxism around 21% and awake bruxism around 23% [1]. Bruxism is a behavior associated with masticatory muscle activity during sleep or wakefulness, characterized by repetitive, sustained tooth contact and/or bracing or thrusting of the mandible [2]. The prevalence of bruxism is influenced by age, assessment methods (i.e., possible, probable, or definitive diagnosis), and other demographic variables [3]. It becomes less common with age, with an observed prevalence of 3% in older adults [1]. Although often underrecognized, frequent or intense rhythmic masticatory activity associated with bruxism can contribute to dental wear, pain in the jaw muscles or temporomandibular joint, and disruption of normal orofacial function [4, 5, 6].

With increased knowledge about bruxism in recent years, the interest of researchers has shifted from considering it solely a pathology to underlying motor activity causing involuntary teeth grinding and jaw clenching [7]. An increasing body of literature suggests that bruxism (both sleep and awake) is influenced by mechanisms such as neurotransmitter regulation, genetic factors, and physiological states rather than only motor activity associated with the temporomandibular joint. The increased bruxism severity is associated with reductions in serotonin levels and altered autonomic functions [8]. The genetic associations studied between bruxism and sleep apnea have shown polymorphism regarding a similar neurogenetic basis [9, 10]. Bruxism is related to changes in inflammatory markers and hormonal disturbances [11], and correlated with pain perception and general health conditions, reinforcing its multifactorial nature [6]. In 2024, the Standardized Tool for the Assessment of Bruxism (STAB) was published to assess the possible consequences, associated risk factors, etiology, and comorbidities associated with bruxism [12].

Researchers have been exploring different ingenious techniques to understand the prevalence, causes, and potential consequences of bruxism, to enhance the management strategies for bruxers' oral and overall health [13]. Current treatments for bruxism range from behavioral techniques and medications to intraoral devices [14]. The management of bruxism is focused on lifestyle, habits, sleep hygiene, and diet to minimize risk factors rather than purely prevent its consequences [15]. Behavioral therapy includes relaxation exercises and stress management with biofeedback, but it does not provide long‐term efficacy as it requires patient compliance and consistent effort. The medications often provide short‐term relief in pain and muscle relaxation associated with bruxism. However, due to their potential side effects and inconsistency in results, the reliability of findings becomes undermined [16, 17]. Considering the ineffectiveness of these approaches, intraoral devices were designed to provide a more comprehensive treatment approach [18]. Occlusal splints, mouthguards, and biofeedback devices manage and reduce potential long‐term consequences of bruxism. The temporary use of contingent electrical stimulations has been done as a non‐invasive approach to reduce bruxism and protect these types of devices [19]. Furthermore, a range of probable and definitive techniques, such as electromyography (EMG), electroencephalography (EEG), video/audio sleep recordings, and electrocardiography (ECG), are used in sleep studies during polysomnography (PSG) to explore the full scope of bruxism and its intervention strategies [20, 21].

According to the International Classification of Sleep Disorders ICSD‐3, PSG represents the reference standard for sleep bruxism studies [22, 23]. In addition to PSG, EMG or EEG in conjunction with ECG and EOG are recorded solely to assess bruxism. EMG records masticatory muscle activity to analyze RMMA during sleep bruxism. Gul et al. have compared EMG from two masticatory muscles, i.e., temporalis and masseter, at three anatomical positions associated with sleep for bruxism detection [5]. Cid‐Verdejo et al. conducted a polysomnographic study with simultaneous recording from an EMG‐EKG device, Brux‐off, to study sleep bruxism episodes [22]. Ishtiaq et al. have designed an EMG‐based device “Bruxi‐alert” to detect bruxism among other masticatory activities and alert the patients [24]. ECG/EKG is acquired to study stress or arousal states and heart rate variability during bruxism episodes. Though EMG is found to be an effective assessment tool for studying muscle activation patterns associated with bruxism, it only provides localized information. It lacks spatial information about muscle activity across broader areas [25]. Moreover, EEG identifies arousals during sleep stages between rapid eye movement (REM) and non‐rapid eye movement (NREM) to pinpoint when bruxism occurs. Michalek‐Zrabkowska et al. recorded EEG and EOG using an eight‐channel system, following the International 10–20 system [21]. The effect of phenotypes of bruxism activity during different sleep stages has been studied using EEG during video PSG, which yielded that there is no particular effect of SB on sleep duration, efficiency, latency, and disturbances leading to arousal from sleep [26]. Since EEG is a complex system, Wang et al. proposed a single‐channel EEG bruxism detection system at the C4P4 location [27]. EOG recordings with EEG are done to observe eye movements during the REM and NREM stages during sleep bruxism episodes. EEG offers high temporal resolution to record electrical activity from the brain's cortical surface, but provides limited spatial resolution to accurately localize the brain activity in specific areas accurately [28]. Over and beyond, EMG and EEG are highly susceptible to motion artifacts and electrical noises from external sources, which impact signal quality. Furthermore, these current diagnostic approaches rely on peripheral markers or labor‐intensive protocols such as polysomnography. In contrast to the mentioned subjective muscle and brain assessment techniques, functional near‐infrared spectroscopy (fNIRS) provides excellent spatial resolution with minimized sensitivity to motion artifacts and electrical noises, which has still not been explored to study brain and muscle activities associated with bruxism.

In recent years, fNIRS has become an effective non‐invasive tool for brain studies, focusing on cortical activity related to cognitive, sensory, and motor tasks. fNIRS is used not only for responses to brain activation but also for muscle activation. Researchers have shown that EMG and fNIRS correlate in dynamic activities [29, 30]. It provides insights into how the brain responds to different muscle contractions [31]. fNIRS can identify the precise motor cortex regions and other related areas that are active during certain motor activities [32]. The neurological basis for bruxism involves the masticatory muscles, temporal, and masseter, and comes from the primary motor cortex (M1) [33]. In correspondence to this, jaw‐clenching [34] and chewing activity [35] have been detected using fNIRS and NIRS respectively. Once the motor activity is monitored using fNIRS, machine‐learning algorithms are employed to detect the respective movements [36, 37]. Given that bruxism involves involuntary jaw clenching during sleep, fNIRS provides a unique opportunity to explore how the brain regulates these actions through hemodynamic changes, offering insights that are less accessible with other methods.

In this research, functional near‐infrared spectroscopy (fNIRS) is used to investigate cortical activity related to motor activities of masticatory muscles and jaw movements associated with simulated bruxism. The novelty of this research is to explore the feasibility of fNIRS as a proof‐of‐concept tool for evaluating hemodynamic patterns for bruxism‐like jaw movements in the motor cortex. The study aims to employ fNIRS to differentiate jaw movement activity by mimicking bruxism among other common jaw movements, using healthy participants as a controlled simulation paradigm. Once the fNIRS data related to the cortical activity of the motor cortex is collected, hemodynamic assessments are done. Lastly, machine learning classifiers are used to classify jaw clenching/teeth grinding associated with bruxism among other masticatory muscle activities of chewing and talking. In this way, the neurophysiological basis of bruxism is explored for further treatments and interventions by objective, scalable, and hemodynamic assessment tools for bruxism.

## 2 Methodology

**FIGURE 1:**
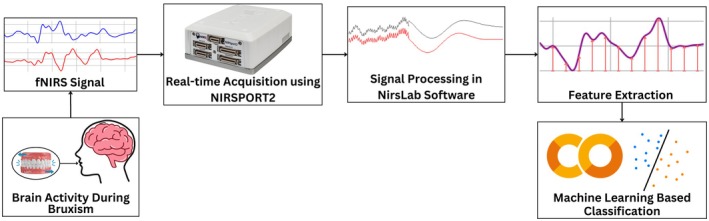
The overall methodology of the research, illustrating fNIRS acquisition from subjects performing bruxism activity using the NIRSport2 system, followed by signal processing, feature extraction, and machine learning‐based classification of masticatory muscle activities.

The overall methodology of the research is shown in Figure 1. The fNIRS functional Near‐InfraRed Spectroscopy data are recorded from subjects performing masticatory muscle activities using the NIRSport2 Acquisition System. Once the signal of interest was acquired and pre‐processed, relevant features were extracted. Then classification among different motor activities associated with masticatory muscles was done to classify bruxism. The detailed methodology of the research and the results obtained are discussed in detail in the remaining sections.

### 2.1 Data Acquisition

The data was acquired from ten healthy subjects. The subject selection criterion was intended to balance genders. Hence, 5 males and 5 females were trained to perform teeth grinding/jaw clenching activity. Each subject's data was recorded in 3 trials. The subjects' inclusion and exclusion criteria were defined based on health conditions and physiological variables of blood pressure, heart rate variability, and oxygen saturation. The inclusion and exclusion criteria are mentioned in Table 1.

**TABLE 1:** Inclusion and exclusion criteria for the experiment.

Sr. #	Parameter	Criterion
*Inclusion criterion*		
1	Blood pressure	Systolic pressure 90–120 mm of Hg
		Diastolic pressure 60–80 mm of Hg
2	Oxygen saturation	Above 95%
3	Heart rate	60–100 bpm beats per minute
4	Caffeine intake	No caffeine intake before 6 h
5	Adequate sleep	At least 6–8 h of sleep before the experiment
6	Hair condition	No oily/greasy hairs
7	Self‐reported bruxism	No self‐reported bruxism
*Exclusion criterion*		
8	Willingness	Unwilling to participate
9	Medications	Under anti‐psychotic or neuromuscular medication
10	Missing teeth	More than two missing teeth per quadrant (excluding third molars)

The subjects were younger adults aged 18–25 with a mean age of 21.5 ± 2.45 years. The subjects had no prior history regarding the diagnosis of sleep or awake bruxism. The literature supports the use of simulated or controlled motor paradigms in healthy individuals to establish the feasibility of a novel approach before moving to clinical populations [38, 39, 40]. This selection choice was intentionally designed as a first step in the exploratory nature of our study to investigate the feasibility of fNIRS in detecting jaw patterns associated with bruxism among other daily activities involving jaw movements of talking and chewing. Further, it was ensured that none of the subjects had any history of neurophysiological or cardiovascular diseases, as both tend to alter the neural activation patterns and associated hemodynamic changes during fNIRS data acquisition. Informed consent was obtained from the subjects as per the Declaration of Helsinki. The subjects were instructed regarding the motor activities, followed by their written consent to participate in the experiment. The subjects were seated in a dark room with proper support to avoid any unnecessary movements, to avoid motion artifacts in the data. The experiment was recorded in a dark room to minimize external optical interference, as external light sources (sunlight, room lights) emit wavelengths that overlap with infrared light used in fNIRS. These stray lights may enter the detectors and introduce artifacts, lowering the signal‐to‐noise ratio. The experiment was approved by the institutional review board of the Biomedical Engineering Department, Air University.

### 2.2 Experiment Configuration and Paradigm

**FIGURE 2:**
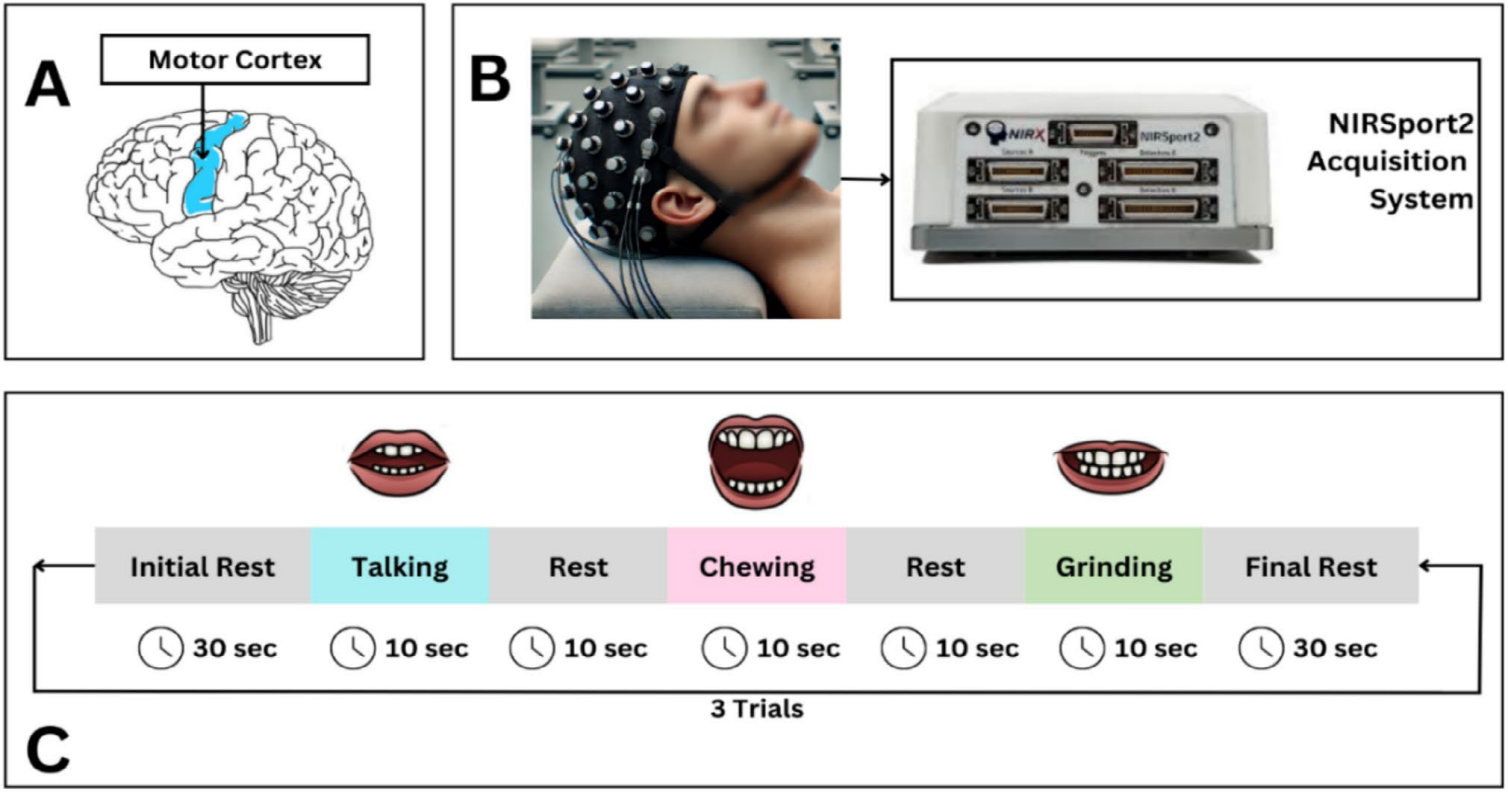
The data of bruxism was recorded from 10 subjects in 3 trials. The subjects were lying in the supine position with the fNIRS cap placed on their heads. Subfigure A shows the region of interest for data acquisition, i.e., motor cortex. Subfigure B shows the acquisition system, NIRSport2, for functional near‐infrared spectroscopy. Subfigure C demonstrates the experimental paradigm followed to record the experiment in 3 successive trials.

The region of interest (ROI) of the brain was first determined to capture the relevant data for bruxism in controlled simulated conditions. The choice of region of interest depends upon the cognitive, sensory, motor, auditory, and visual processes and tasks under the experimental investigation. Since bruxism is associated with rhythmic masticatory muscle activity (RMMA), the motor cortex was selected as the region of interest. The RMMA involves repeated contractions associated with jaw movement, typically teeth grinding (side‐to‐side motion) and jaw clenching (up‐and‐down motion). These movements are related to brain activity in the motor cortex region of the frontal lobe. Hence, the bruxism data from the motor cortex was acquired using functional near‐infrared spectroscopy, as shown in Figure 2A.

**FIGURE 3:**
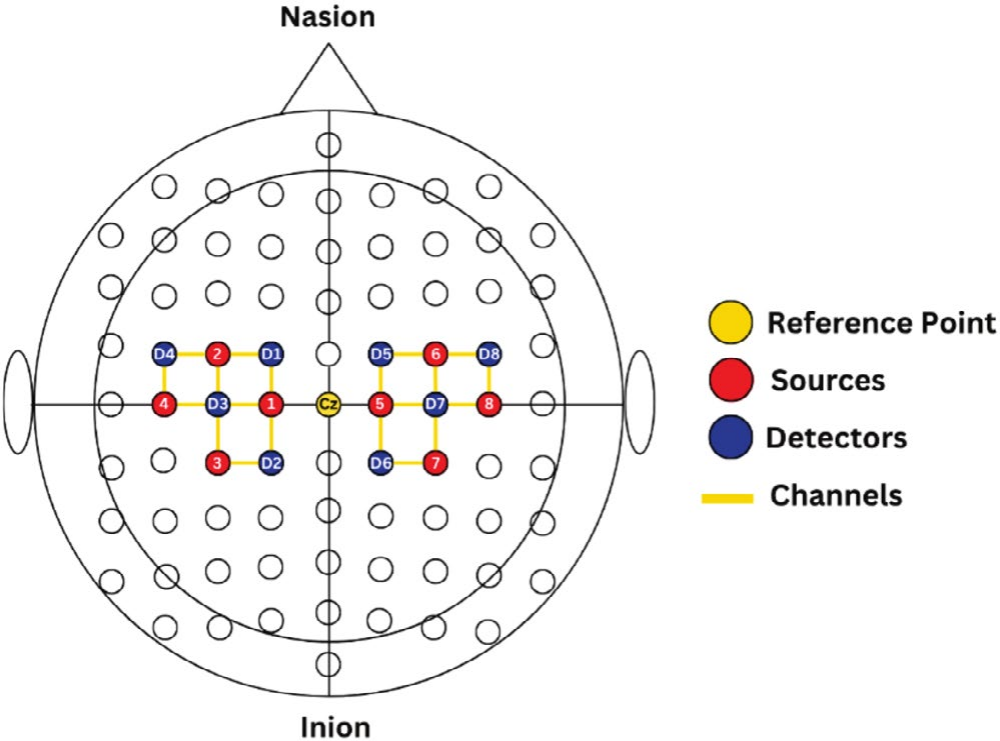
Optode placement layout over the motor cortices using the international 10–20 system as a reference. The central landmark Cz is located at the intersection of the midline (from nasion to inion) and the coronal line (from left to right preauricular points). Each optode in this layout is separated by a distance of 3 cm.

The acquisition system used was a continuous wave NIRSport2 fNIRS system by NIRx Medical Technologies, Germany, as shown in Figure 2B. The NIRSport2 used in this experiment operates on two wavelengths, 760 and 850 nm, with a sampling frequency of 10.1725 Hz. The experimental montage was designed with a total of 16 optodes, placed bilaterally on the motor cortex region, with 8 optodes on the left and right hemispheres each. Among these 8 optodes, 4 were near infrared (IR) light sources and 4 were near IR light detectors. The optode placement is done according to the 10–20 EEG system for the localization of ROI. The channel distance was kept at 3 cm from each optode [41]. A total of 20 channels were established by the defined montage over the motor cortex of the NIRSport2 system, with 10 channels on each side, i.e., left and right hemispheres, as shown in Figure 3.

**FIGURE 4:**
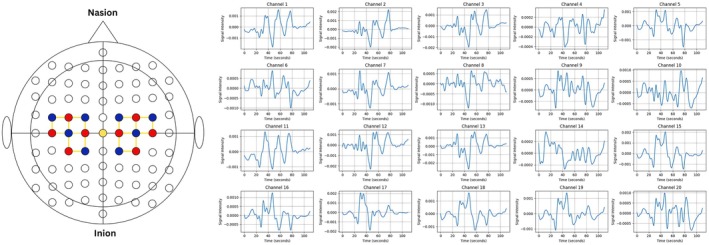
The Experimental Configuration for recording fNIRS during motor activities associated with bruxism, showing the establishment of 20 channels, 10 on each side of the left and right hemispheres.

The data acquisition software Aurora by NIRx Technologies was used, which provided a full suite of tools for data acquisition with NIRSport2. It was connected via Wi‐Fi to the NIRSport2 System. The channels were established and monitored using Aurora software by NIRx Technologies, with yellow lines showing excellent channels between the source and detector. The establishment of channels between sources and detectors in the NIRSport2 system is done with the help of the automated signal optimization algorithm of Aurora software that ensures optimal signal quality before data acquisition is started. The fNIRS cap with the specified montage is placed on the subject, and a test is run to check the channel establishment between the source and detector as excellent or bad, shown by yellow or red lines respectively. The data corresponding to 20 channels from the motor cortex region is then recorded, which displays the 20‐channeled fNIRS data of concentration changes in real time as shown in Figure 4.

The data was acquired following the experimental paradigm shown in Figure 2C. The paradigm was designed in 2023.2.3 software. The participants were instructed to perform bruxism‐like activity of jaw clenching and teeth grinding and other masticatory muscle activities. The experiment was performed in 110 s. The subject was asked to lie in the supine position. The fNIRS cap was placed to cover the motor cortex region by optodes placed on the cap. An opaque black flexible cap was placed over the fNIRS cap to block any ambient light. The subjects were instructed to avoid deliberate movements which may add motion artifacts to the acquired data from the motor cortex. The paradigm was designed with an initial and final rest of 30 s, with motor activities and an intermediate rest, each of 10 s. The initial and final rest is included to establish a baseline condition for stabilized hemodynamic response. On the other hand, intermediate rests re‐establish the baseline condition after each motor activity and avoid saturation in brain activity by continuous movements of different types (chewing, talking, teeth grinding). The motor activities were included to differentiate among masticatory muscle movements of talking, chewing, and teeth grinding. Each of the 10 subjects performed 3 trials of this experiment, resulting in 10 × 3 = 30 data files for each trial of all subjects.

Once the data was acquired, it was preprocessed in MATLAB‐based NirsLab software.

### 2.3 Data Pre‐Processing in NirsLab

The raw data from the Aurora software was imported to the NirsLabv2019.04 in .nirs format. The NirsLab software was used for signal pre‐processing, analysis, and visualization of hemodynamic responses associated with the experiment. The event markers were added from the PsychoPy file created for the experimental paradigm. The data quality feature of the software was used to check good and bad channels to make the channel selection process convenient. Then frequency filters were applied to filter the fNIRS data for all trials of each of the ten subjects. The bandpass filter was selected with a roll‐off width of 10%, a low cut‐off frequency of 0.01 Hz, and a high cut‐off frequency of 0.1 Hz [42]. This helps to filter the high‐frequency physiological noises from the heartbeat, respiratory signals, and instrument noise via the low‐pass filter and low‐frequency noises of baseline drift via the high‐pass filter. The signal was further analyzed for any spikes that deviate significantly from the normal fNIRS signal. Other than frequency filters, the Spike Artifact Detection feature in NIRSlab was used. It works by using a derivative threshold‐based algorithm and removes the artifacts—typically caused by facial movement or optode displacement. The spike artifact detection was used over the motor segments of talking, chewing, and grinding to preserve continuity without introducing bias.

After filtration, optical preprocessing was done using modified Beer–Lambert Law (MBLL). The pre‐defined hemodynamic parameters for Beer–Lambert Law were used to compute the changes in oxyhemoglobin HbO and de‐oxyhemoglobin HbR. The differential path factor was selected as predefined in the software, with wavelength 1 of 7.25 and wavelength 2 of 6.38. The amount of light absorbed is influenced by the concentration of hemoglobin in its oxygenated and deoxygenated states. Using Equation (1), the changes in ΔHbO and ΔHbR concentrations were measured:

(1) $$\left(\begin{array}{c} ∆{\left(\mathrm{HbO}\right)}_{t}\\ ∆{\left(\mathrm{HbR}\right)}_{t} \end{array}\right)=\frac{{\left[\begin{array}{cc} \text{σHbO}\left(\varphi_{1}\right) & \text{σHbR}\left(\varphi_{1}\right)\\ \text{σHbO}\left(\varphi_{2}\right) & \text{σHbR}\left(\varphi_{2}\right) \end{array}\right]}^{-1}\left[\frac{∆O_{D}\left(t,\varphi_{1}\right)}{∆O_{D}\left(t,\varphi_{2}\right)}\right]}{\mathrm{Lxd}}$$

**FIGURE 5:**
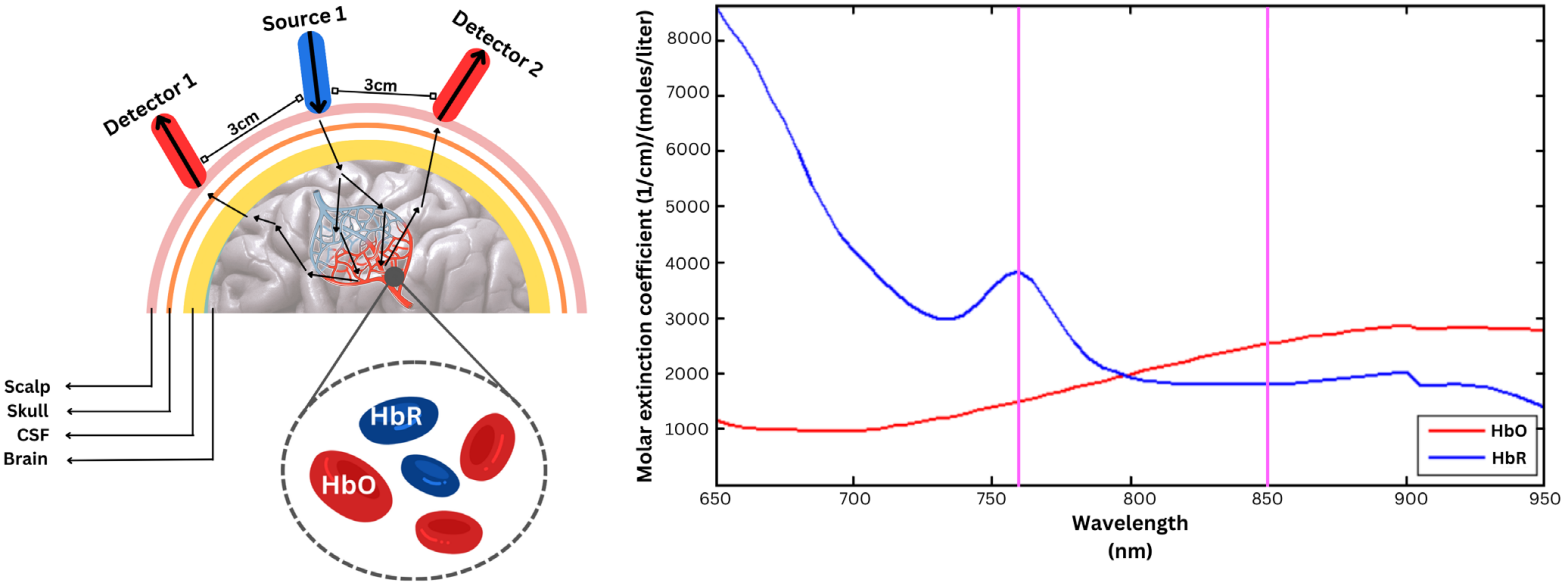
The pictorial illustration of the mechanism of functional‐near infrared spectroscopy, involving the IR light absorption into the brain to record the changes in concentration of oxy and deoxyhemoglobin. The source emits the IR light which, after absorption, is detected by detectors with an inter‐distance of 3 cm between each source and detector. The hemodynamic response plot from NirsLab of masticatory activities, with events marked for the segment of bruxism, is associated with teeth grinding. The hemodynamic response computes the change of oxy‐hemoglobin and deoxy‐hemoglobin, illustrating the maximized change in concentration of oxy‐hemoglobin as the subject performs bruxism, demonstrating the activation of the motor cortex region.

The change in the absorbance of infrared light over time provides information about changes in the concentration of HbO and HbR and provides key indicators of neural activities in rest and motor segments of chewing, talking, and grinding over the region of the motor cortex. The absorption spectrum was then plotted following W.B. Gratzer. This hemodynamic response is shown in Figure 5.

**FIGURE 6:**
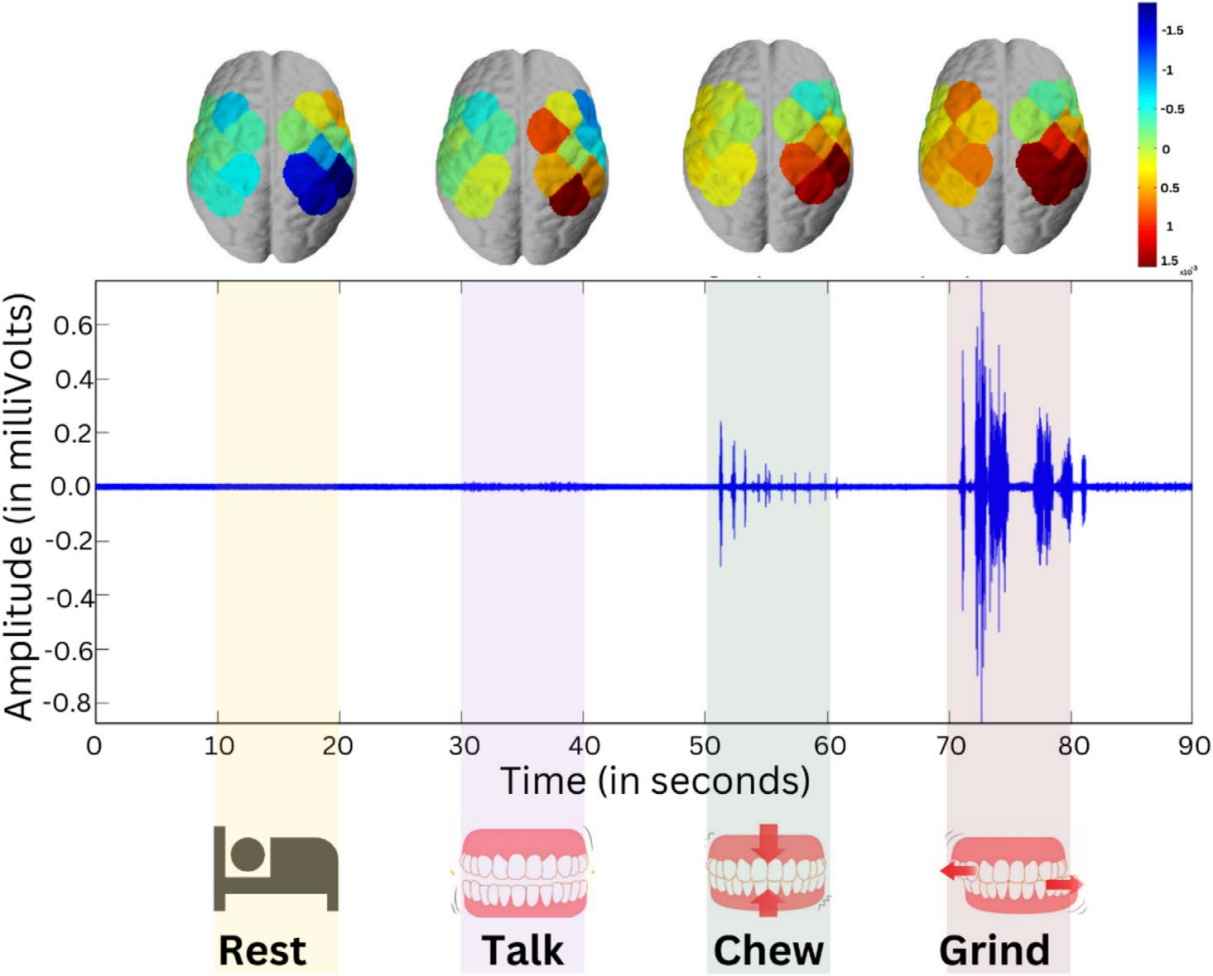
The brain activation maps corresponding to electromyogram to record muscle activity from the temporalis muscle, for the activities of resting, talking, chewing, and grinding to assess the hemodynamic states associated with the change in oxyhemoglobin across the motor cortex region.

Once the pre‐processing was done, the spatial activation maps were computed. The topographic maps provided a pictorial representation of the cortical activation across the motor cortex for the experiment associated with the change of oxyhemoglobin HbO across segments of rest, talking, chewing, and teeth grinding. This allows the comparative analysis of the activated brain region of the motor cortex in different motor activities. The brain activation maps corresponding to the electromyogram EMG of the temporalis muscle are illustrated in Figure 6. The electromyogram from the temporalis muscle was recorded simultaneously with fNIRS to identify and validate the motor activity of talking, chewing, and grinding. The temporalis muscle was selected as it records the masticatory activities and helps in identifying the occurrence of these activities [5, 24]. The fNIRS, along with EMG of the temporalis muscle, helped in tracking the duration and intensity of muscle activation. It helped in the validation of the occurrence of masticatory activities properly. The electromyogram illustrated shows the muscle activation, which is least in talking, intermediate in chewing, and maximum during teeth grinding and jaw clenching. Since the subjects were non‐bruxers, they were trained to perform the activity of bruxism by clenching their jaws and teeth grinding; EMG provided an assessment of muscle forces and activation patterns in the segments of grinding.

The processed data for HbO was exported in .csv format for pre‐processing and feature extraction followed by machine learning‐based classification of bruxism.

### 2.4 Feature Extraction

The pre‐processed 20 channeled data from NirsLab was imported to Google Colaboratory for feature extraction and machine learning, which provides free access to computing resources for machine learning tasks. The 20 channeled data was spatially averaged before the feature extraction process. The optode placement is distributed across the motor cortex; hence, the spatial average was obtained for all the channels rather than focusing on selected and optimized channels. The spatial average was obtained to ensure the entire ROI is represented to capture the overall trend in oxygenation levels. This also simplifies the analysis without loss of essential information in the data. The spatial average was taken using the formula described in Equation (2):

(2) $$\text{Spatial Average}\left(t\right)=\frac{1}{N}\sum_{k=0}^{N}{\text{Signal}}_{k}\left(t\right)$$

where *N* = 20.

The average data was segmented according to different motor activities, including initial rest, talking, intermediate rest, talking, intermediate rest, chewing, intermediate rest, talking, and final rest. The segmented data was labeled into four classes, i.e., Label 0 for Rest, Label 1 for Bruxism (Teeth Grinding), Label 2 for Talking, and Label 4 for Chewing. This approach helps to minimize inter‐channel variability and enhance signal reliability, in line with recent recommendations for optimizing fNIRS data quality and reproducibility.

**FIGURE 7:**
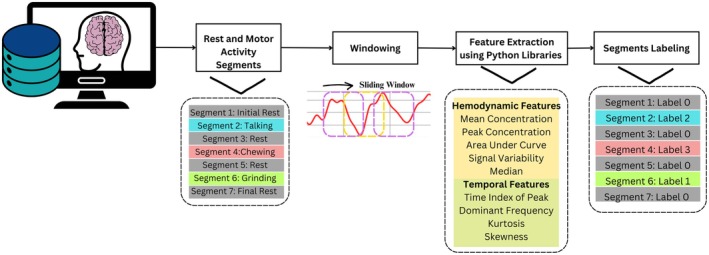
The overall flow for the data segmentation, labeling, and feature extraction of fNIRS data.

The statistical features were extracted over the segmented data following the sliding window approach, with an overlapping window of size of 10. A total of 12 features were extracted, including mean, root mean square, peak, area under the curve, standard deviation, kurtosis, skewness, low‐frequency oscillations, very low‐frequency oscillations, cardiac frequency band, respiratory frequency band, and dominant frequency. Among these features, the statistical features mean, root mean square, standard deviation, kurtosis, and skewness were extracted to provide information about signal distribution and variability. Other than that, hemodynamic features such as the area under the curve and peak describe blood flow and oxygenation dynamics in HbO and HbR. Lastly, frequency domain features analyze oscillatory patterns and physiological influences. The resulting extracted features file contained 2369 samples, further used for machine learning classification. This flow is shown in Figure 7.

### 2.5 Machine‐Learning Based Classification

The machine learning pipeline for this research is illustrated in Figure 6. Once the feature set was extracted, it was analyzed and preprocessed to look for any missing values and normalization. Instead of a single train‐test split, k‐fold cross‐validation was applied to ensure that the model does not learn patterns specific to a single train‐test split, leading to overfitting in the trained machine learning classifiers. The value of *k* was set to be 10. After normalization and cross‐validation, the feature set was optimized to reduce computation time by selecting the most relevant features to increase the efficiency of machine learning models for the assigned classification task of bruxism. Among several approaches to feature optimization, three feature optimization techniques were employed, namely, feature selection, feature reduction, and feature importance [43, 44]. Each technique works differently but shares a common goal of building efficient machine‐learning models with less computation time. Feature selection is the technique of choosing the most relevant features and removing redundant features by employing methods including wrapper and filter methods. Among wrapper methods, the technique of recursive feature elimination (RFE) was employed. The estimator was chosen to be gradient boosting XGBoost classifier, which identified the relevant features and iteratively removed the least important features. Mutual information (MI), a filter method, was used for feature selection by selecting the features carrying the highest information about the predicting variable. Once mutual information scores for all the features were computed, a threshold was defined to include the features with high MI scores. To optimize the features using the technique of feature importance, tree‐based methods were employed. The feature importance scores were computed using the Gradient Boosting XGBoost classifier, which works by assigning feature importance based on how frequently a feature is used in classification. Lastly, for feature reduction, principal component analysis (PCA) was done, which transforms the data into lower dimensions while retaining the information contained in features. The principal component analysis was done to retain the maximum variance in data of 95%. Like PCA, linear discriminant analysis (LDA) was also performed as it focuses on class separability in classification problems rather than retaining variance.

**FIGURE 8:**
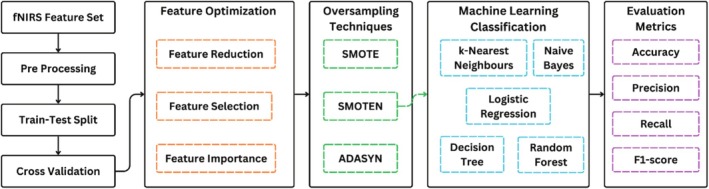
Machine Learning Pipeline for classification of different masticatory muscle activities to detect bruxism, including feature optimization, over‐sampling techniques, and training and evaluating machine learning classifiers.

Once the machine learning models were pipelined for computational efficacy, the class imbalance in motor activity segments was handled. The designed paradigm includes multiple segments of rest causing 80 s of rest class compared to 10 s of motor activities each, which may lead to biases in the classification results. To overcome this issue, machine learning models were compared over different synthetic oversampling techniques to ensure that each class contributes equally to model learning, improving generalization. The techniques of Synthetic Minority Oversampling Technique (SMOTE), Synthetic Minority Over‐sampling Technique for Nominal features (SMOTEN), and Adaptive Synthetic Sampling (ADASYN) were used to generate synthetic samples of minority classes, improving the class balance across all four classes [45]. The evaluation metrics to evaluate the machine learning models included accuracy, precision, recall, and F1‐score. The machine learning models employed were k‐nearest neighbors, logistic regression, Naïve Bayes, decision trees, and random forest. The models were compared, and the most effective model was selected for the classification of bruxism using fNIRS data. The machine learning pipeline is illustrated in Figure 8.

## 3 Results

This section presents the results of machine‐learning‐based detection of rhythmic masticatory muscles' activity associated with simulated bruxism.

### 3.1 Assessment of Hemodynamic States

**FIGURE 9:**
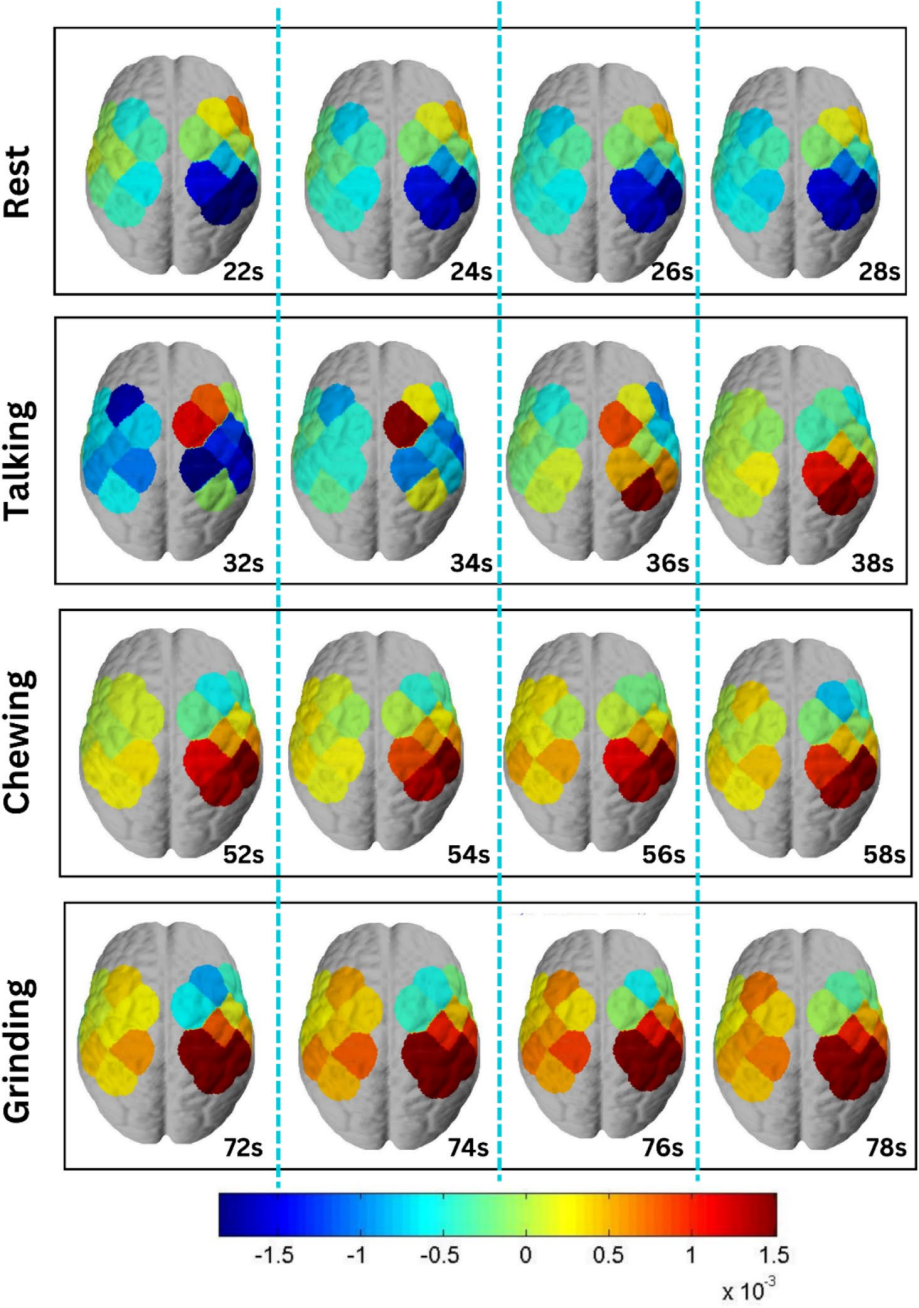
Hemodynamic assessment of brain activation maps during rest and masticatory activities of talking, chewing, and grinding at intervals of 2 s for a total of 10‐s segments, comprising 20 to 30 s of initial rest, 30 to 40 s of talking, 50 to 60 s of chewing, and 70 to 80 s of grinding.

The hemodynamic assessment through brain activation maps was done through NirsLab Software as discussed in Section 2.3 of the methodology. The hemodynamic assessment allows us to visualize the change in concentration of oxyhemoglobin across the areas and divisions of the motor cortex. Since the experiment performed masticatory activities of talking, chewing, and grinding, which all originate from the motor cortex. To differentiate between these motor activities, the hemodynamic activation maps were plotted at intervals of 2 s for rest, talking, chewing, and grinding as shown in Figure 9.

### 3.2 Features Optimization

The grand‐averaged results across all subjects from feature optimization are tabulated in Table 2. The machine learning models were evaluated for train and test accuracy, precision, recall, and F1‐score before and after feature optimization techniques. The feature optimization techniques applied include mutual information, recursive feature elimination, tree‐based feature importance, principal component analysis, and linear discriminant analysis. The train and test accuracies were computed to check for overfitting in the machine learning models before and after feature optimization.

**TABLE 2:** The accuracies (in percentages) of different machine learning classifiers before feature optimization and after feature optimization techniques, including principal component analysis, mutual information, recursive feature elimination, feature importance, and linear discriminant analysis.

	Model	Train accuracy	Test accuracy	Precision	Recall	F1‐score
Before feature optimization	kNN	78.36	71.87	64.77	71.87	66.70
	Logistic Regression	72.46	72.33	57.22	72.33	62.65
	Naïve Bayes	63.74	64.39	62.47	63.49	62.19
	Decision Tree	73.43	73.17	60.48	73.17	62.92
	Random Forest	74.69	74.84	72.70	74.84	66.98
Principal component analysis	kNN	77.57	71.90	64.39	71.90	66.33
	Logistic Regression	72.65	72.79	57.91	72.79	62.78
	Naïve Bayes	70.73	70.83	60.00	70.83	62.79
	Decision Tree	73.33	72.92	63.21	72.92	62.51
	Random Forest	75.17	74.03	68.42	74.03	65.08
Mutual information	kNN	78.10	71.92	64.80	71.92	66.85
	Logistic Regression	72.56	72.68	57.65	72.68	62.80
	Naïve Bayes	69.97	69.93	61.37	69.93	63.63
	Decision Tree	73.43	73.17	60.48	73.17	62.92
	Random Forest	77.01	75.31	73.37	75.31	67.79
Recursive feature elimination	kNN	75.66	74.37	74.21	74.37	66.03
	Logistic Regression	73.18	73.04	60.09	73.04	62.71
	Naïve Bayes	69.64	69.80	58.49	69.80	63.16
	Decision Tree	72.45	72.35	57.23	72.35	62.63
	Random Forest	78.57	73.01	67.36	73.01	68.33
Feature importance	kNN	79.05	73.58	68.40	73.58	69.16
	Logistic Regression	72.36	72.22	56.94	72.22	62.45
	Naïve Bayes	66.67	66.40	63.42	66.40	63.35
	Decision Tree	73.27	73.02	59.75	73.02	62.80
	Random Forest	75.95	74.74	73.80	74.74	66.96
Linear discriminant analysis	kNN	83.04	76.32	73.26	76.32	73.69
	Logistic Regression	72.50	72.38	66.69	72.38	62.87
	Naïve Bayes	71.06	71.26	60.83	71.26	63.47
	Decision Tree	73.25	73.11	62.15	73.11	62.44
	Random Forest	76.05	74.70	70.90	74.70	66.47

Once the feature optimization techniques were applied, the difference was calculated for all classifiers before and after feature optimization techniques, including principal component analysis, mutual information, recursive feature elimination, feature importance, and linear discriminant analysis. The calculated differences are tabulated in Table 3.

**TABLE 3:** The difference in accuracies of machine learning classifiers by applying feature optimization techniques.

Feature optimization	k‐NN	Logistic Regression	Naïve Bayes	Decision Tree	Random Forest
Principal component analysis (PCA)	−0.0079	+0.0019	+0.0699	−0.0010	+0.0048
Mutual information	−0.0026	+0.0010	+0.0623	0.0000	+0.0232
Recursive feature elimination (RFE)	−0.0270	+0.0072	+0.0590	−0.0098	+0.0388
Feature importance (Tree‐based)	+0.0069	−0.0010	+0.0293	−0.0016	+0.0126
Linear discriminant analysis (LDA)	+0.0468	+0.0004	+0.0732	−0.0018	+0.0136

**FIGURE 10:**
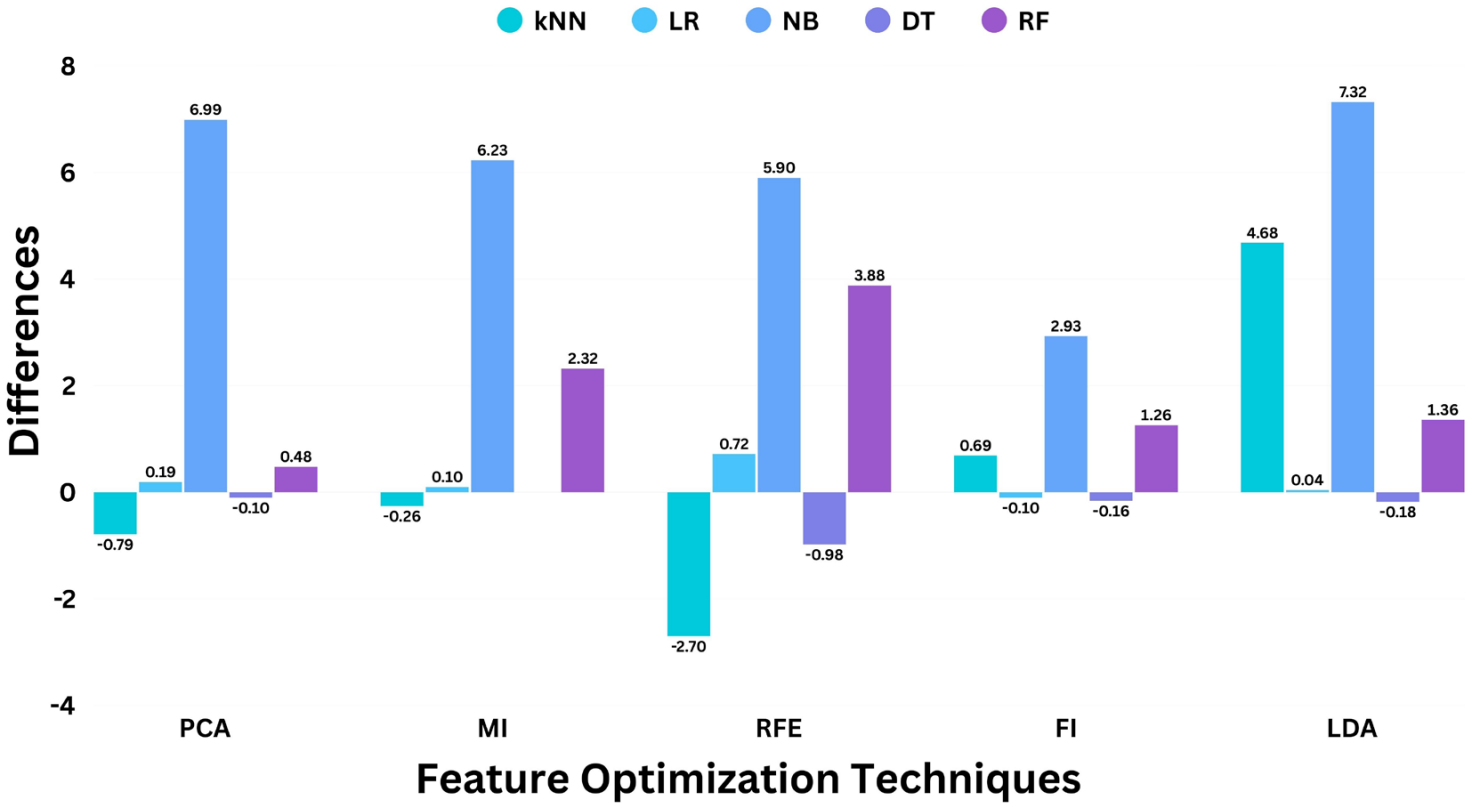
The difference in accuracies in percentages of machine learning classifiers, k‐nearest Neighbors, Logistic Regression, Naive Bayes, Decision Tree, and Logistic Regression with and without feature optimization techniques.

The difference in grand‐averaged accuracies in percentages of machine learning classifiers before and after feature optimization is illustrated in the bar chart in Figure 10.

### 3.3 Class Imbalance Handling

**FIGURE 11:**
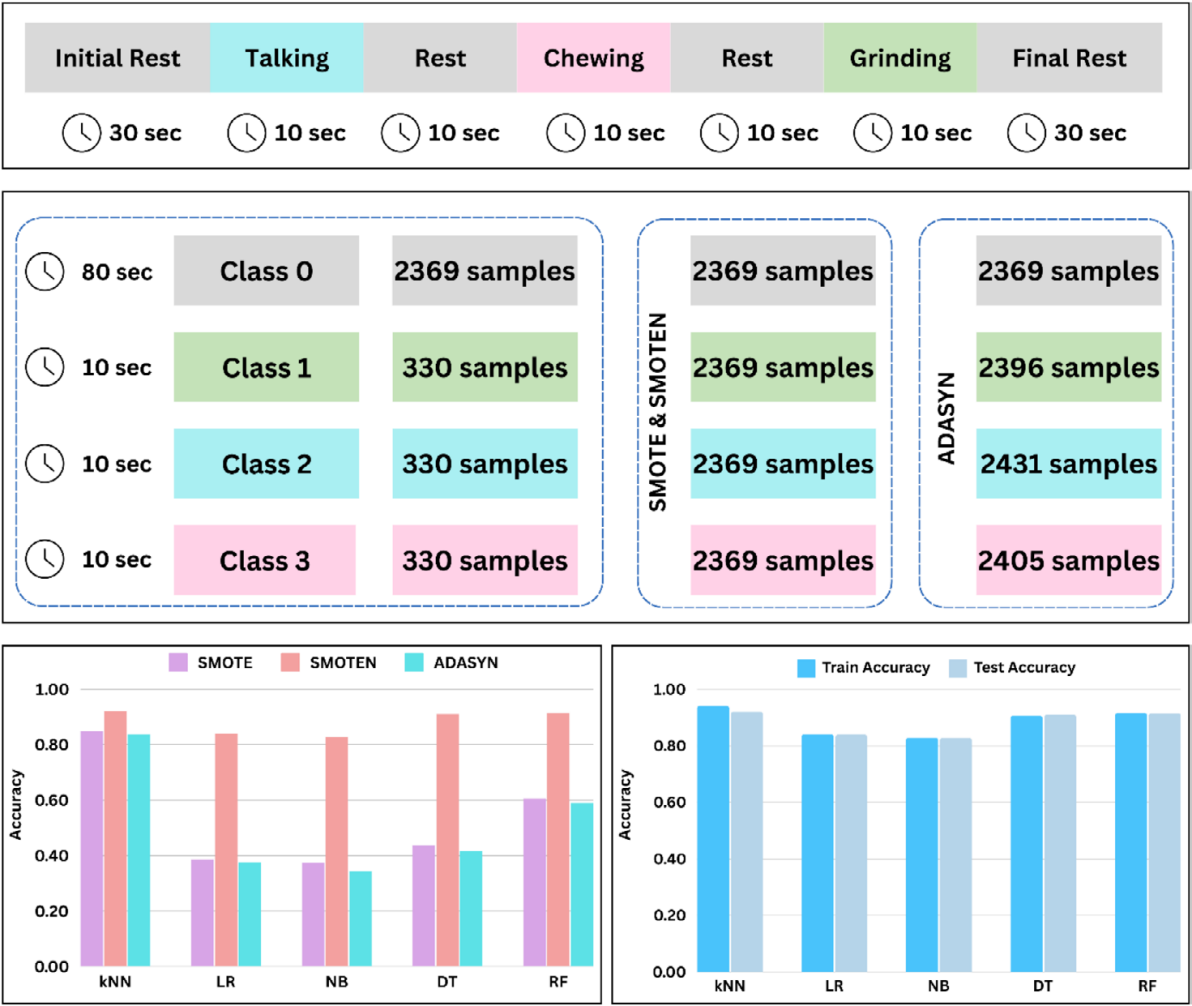
The number of samples in each class of the experiment comprising segments of rest, talking, chewing, and grinding before and after oversampling techniques, with the accuracy comparison of SMOTE, SMOTEN, and ADASYN for all machine learning classifiers. The comparison of train and test accuracies for kNN, Logistic Regression, Naive Bayes, Decision Tree, and Random Forest after applying SMOTEN to check for overfitting among the classifiers by studying the differences in train and test accuracies.

Once the features were optimized, the class imbalance between bruxism and non‐bruxism in the dataset was handled. The class distribution initially was such that there were 2369 samples in the rest (Class 0) and only 330 samples in Class 1, Class 2, and Class 3 for bruxism, talking, and chewing, respectively. This imbalance was addressed by oversampling the minority classes, i.e., bruxism (Class 1), talking (Class 2), and chewing (Class 3). Different over‐sampling methods were employed and compared to choose the one with effective results in detecting RMMA associated with bruxism. The three methods were the synthetic minority over‐sampling technique (SMOTE), the synthetic minority over‐sampling technique for nominal (SMOTEN), and Adaptive Synthetic Sampling (ADASYN). The minority class was oversampled to have 2369 samples after applying SMOTE and SMOTEN, and 2396 samples after applying ADASYN in the bruxism class, as shown in Figure 11. After using each technique, the grand‐averaged accuracy across all subjects of machine learning classifiers was obtained, as shown in Table 3.

The comparative analysis of three oversampling techniques was done which showed the best performance by k‐Nearest neighbors when SMOTEN was used. Other than that, bar charts were plotted for the train and test accuracies of SMOTEN among different classifiers since SMOTEN outperformed in the generation of synthetic samples among SMOTE and ADASYN. The train and test accuracies were computed to look for any overfitting in the machine learning classifiers. Since the difference between test and train accuracy was low, it showed that the trained models do not overfit the trained data and perform well on test data.

**FIGURE 12:**
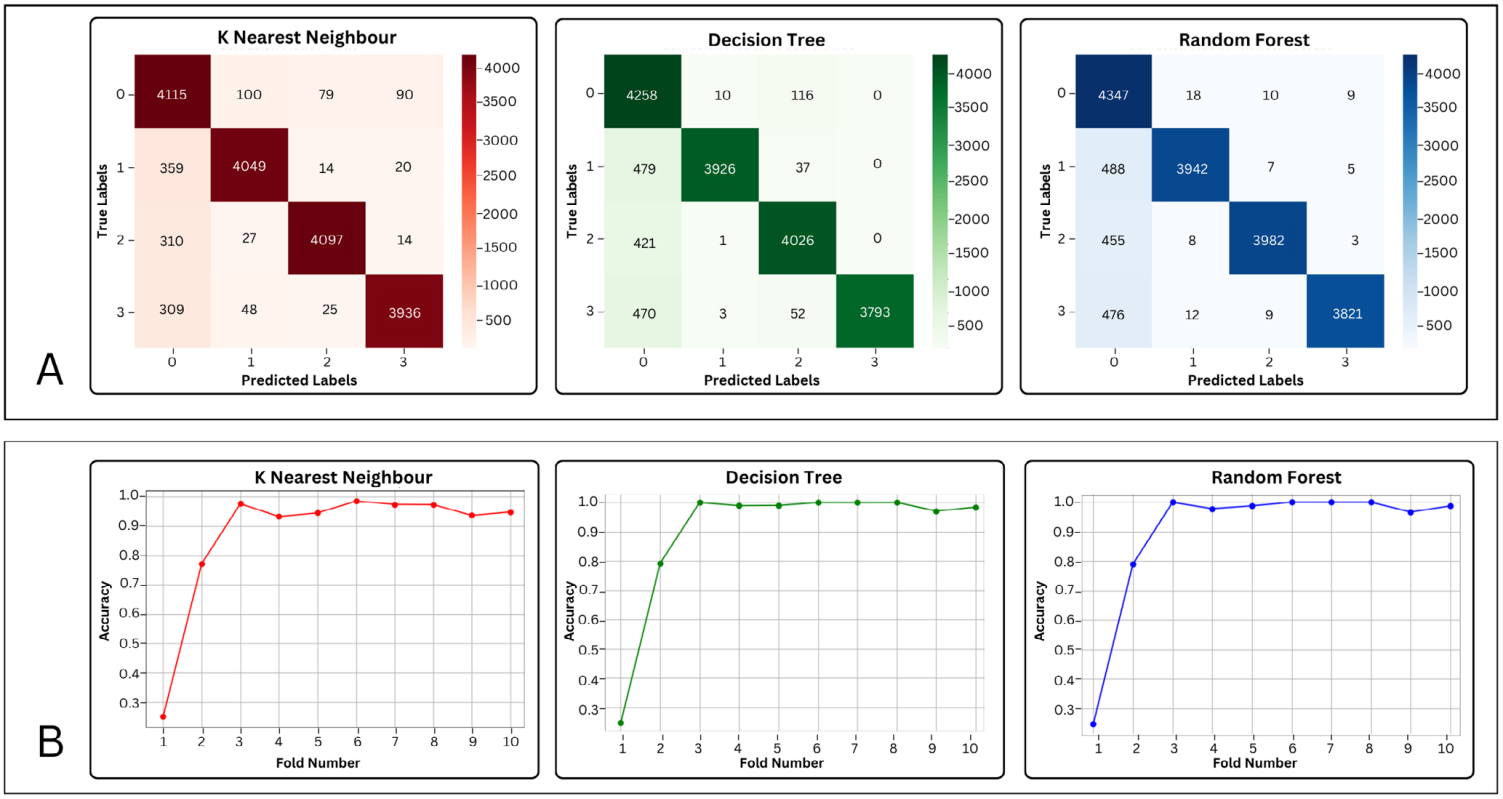
(A) The confusion matrix for K‐Nearest Neighbor, Decision Tree, and Random Forest, (B) K‐fold accuracy curve for K‐Nearest Neighbor, Decision Tree, and Random Forest over 10 folds of cross‐validation.

The confusion matrices and accuracy over 10‐fold cross‐validation were computed for k‐Nearest Neighbors (kNN), Decision Tree (DT), and Random Forest (RF) as they outperformed comparable to each other, achieving accuracies of 92.07%, 90.97%, and 91.46%, respectively. The confusion matrix and cross‐validation accuracy curve of kNN, DT, and RF are given in Figure 12.

### 3.4 Inter‐Subject Accuracy Evaluation

**FIGURE 13:**
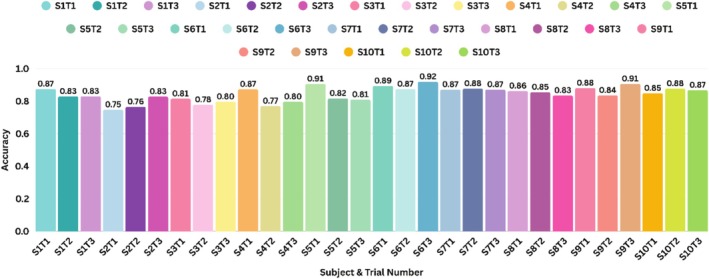
The test accuracy of each subject for each trial using kNN, with LDA and SMOTEN applied, along with 10‐fold cross validation. The tags shown as S1T1 mean subject 1, trial 1, till S10T3, meaning the 3rd trial of the 10th subject.

In addition to grand‐averaged accuracies, after feature selection and oversampling techniques, the accuracies were also computed for each subject as it provides a better understanding of classifier robustness and inter‐individual variability. The accuracy across each individual for each of the three trials is shown in Figure 13.

## 4 Discussion

The cortical activity associated with simulated bruxism in the motor cortex was recorded under a controlled experimental paradigm using functional near‐infrared spectroscopy, as a proof‐of‐concept study that timely detection of bruxism prevents further complications associated with the physical well‐being of bruxers. For this, the rhythmic masticatory muscular activity was recorded and differentiated from other associated masticatory muscle activities, i.e., chewing and talking, which are not‐so‐rhythmic activities. The data recorded using the NIRSport2 acquisition system via Aurora Software was first pre‐processed in NirsLab software. The hemodynamic states were computed on pre‐processed data for rest, talking, chewing, and grinding states at 2‐s intervals, as shown in Figure 9. It was observed that the left hemisphere of the subject was activated more as compared to the right hemisphere due to functional lateralization of the brain [46], indicating the subject is right‐handed. The motor cortex activation varies during different motor activities. During the resting segment, the person is lying in the supine position, which minimizes muscle activity and supports muscle relaxation anatomically. The hemodynamic plots showing active regions of the motor cortex during this segment showed low activity. Some of the active regions shown are due to some subtle muscle activity, such as eye movements, postural adjustments, or involuntary twitches, as the subject was lying awake. In this case, the motor cortex may still send low‐level signals to maintain muscle tone, as shown in the hemodynamic plots of rest segments in Figure 9. Moreover, the subject was given audio stimuli to perform motor activities, and the motor cortex may show transient activations in response to potential movement preparation [47]. With all these considerations kept, the rest segment shows negligible activity in the motor cortex as compared to the motor activities of talking, chewing, and grinding. For the segment of talking, the language center of the brain (Broca's area, Wernicke's area) and the primary motor cortex get activated. The role of the motor cortex in the segment of talking is to control orofacial muscles responsible for the specified motor control of the jaw, tongue, and lips to produce speech. The major contribution is by the language center of the brain in the segment of talking, and hence, the hemodynamic assessment shows the maximum activity in a particular region of the motor cortex. This increased activity is due to the orofacial muscles involved in the process of talking. On the other hand, the segments of chewing and grinding show maximized activation of the motor cortex as compared to the segments of talking and rest. The activated regions of the brain during chewing and grinding are the primary motor cortex (M1), premotor cortex, and supplementary motor area (SMA) [48]. As observed, the activated areas in both segments are the same, though bruxism (grinding) happens involuntarily, but since this experiment took place in a controlled environment, the grinding was performed voluntarily by giving training to subjects. Still, grinding was performed by forceful clenching of the jaw as compared to chewing, which doesn't require force; the activated channels showed more activity in the segment of grinding as compared to chewing [49]. This comparative analysis explains the concentration changes of HbO during different motor activities and resting segments through an illustration of activated areas of the motor cortex, the maximum activation during teeth grinding and jaw clenching, while the absence of motor activity during the segment of rest showed negligible activation of the motor cortex.

Then, twelve features were extracted using MATLAB, and the feature set was optimized to train machine learning classifiers. The feature set was optimized before training the machine learning models. The feature optimizers were evaluated with 10‐fold cross‐validation using grand‐averaged train accuracy, test accuracy, precision, recall, and F1 score for five machine learning classifiers. These models were first evaluated without optimization of features, with a complete feature set of 12. This gave the grand‐averaged test accuracy of 71.87% with k‐NN, 72.33% with LR, 64.39% with NB, 73.17% with DT, and 74.84% with RF, as tabulated in Table 2. The features were optimized using methods of feature reduction, selection, and importance. For feature reduction, PCA and LDA were used to optimize the features. The models were trained for reduced features to keep a maximum variance of 95% when doing principal component analysis. In the case of LDA, maximum class separability between four classes for 12 features was retained by reducing features with the limitation of the number of classes and feature set. The maximum test accuracy achieved was found to be 77.57% for k‐NN and 83.04% for k‐NN for PCA and LDA, respectively. Next, feature selection was done to optimize features using recursive feature elimination (RFE) and mutual information (MI). For RFE, the base model was selected as the gradient‐boosting classifier to evaluate features by learning patterns of data at a learning rate of 0.1. The RFE then recursively removed the least important features till four features remained. The test accuracy achieved using RFE was 74.37% with k‐NN. Next, mutual information was deployed to find k‐best features by defining the scoring function as mutual information from built‐in libraries of Python. The test accuracy achieved was 75.31% with the RF classifier. Lastly, features were optimized using tree‐based methods in which the extreme gradient boosting classifier (XGB) was used to rank features based on their importance in predicting the target variable by calculating the gain. The features were then selected by defining the threshold to include important features in the model training process. This method gave the test accuracy of 76.32% with k‐NN. These feature optimization methods were evaluated for all machine learning classifiers, and then the difference in percentages in test accuracy was computed and tabulated in Table 3 to evaluate the effectiveness of feature optimization in terms of test accuracy. We observed that optimizing the features did not improve the grand‐averaged accuracy of machine learning classifiers significantly. One of the factors contributing is the model overfitting, but it was avoided, as 10‐fold cross‐validation was applied during the train‐test split before training the machine learning classifiers. Also, train and test accuracy were computed to find the overfitting in performance by evaluating if there is high accuracy on the training set but poor performance on the test set. If there exists a small gap between train and test accuracy, the model may have essentially memorized the training data rather than learning generalizable patterns. The train and test accuracy, as tabled in Table 2 showed the gap is small between train and test accuracy, which means machine learning models are not too complex to overfit. Hence, if the model is not overfitting and optimizing the features does not improve the accuracy, the data was pre‐processed further to improve the quality and quantity of data.

The four classes in the dataset are named: rest as Class 0, grinding/bruxism as Class 1, talking as Class 2, and chewing as Class 3. Since the experimental paradigm has multiple rest segments, which are essential to have when recording brain cortical activity, as it establishes the baseline signal between different motor activities, this led to an increased sample in the rest class, as it contained 80 s data. On the other hand, each motor activity was recorded for 10 s. The samples in each class were computed as shown in Figure 11. This class imbalance was handled by oversampling of the minority classes, i.e., classes 1, 2, and 3. Since the highest accuracies and maximum increased difference before and after feature optimization were observed by Linear Discriminant Analysis, for all classifiers, the machine learning pipeline was updated to apply oversampling along with LDA. The technique of oversampling was preferred to improve model generalization, leading to a reduced bias toward the majority class, i.e., rest. Compared to oversampling, undersampling the majority class to meet the samples of minority classes, classes 1, 2, and 3 were not considered as it would have reduced the size of the dataset, leading to a loss of information and overfitting. The synthetic (creates synthetic samples for minority classes) rather than random (duplicates the samples for minority class) oversampling techniques were applied and compared, including SMOTE, SMOTEN, and ADASYN. The number of samples before any oversampling technique was applied was 2369 in class 0 (rest) and 330 in the classes of motor activities, i.e., classes 1, 2, and 3. After applying SMOTE and SMOTEN, the minority classes were oversampled to have 2369 samples. This led to equally balanced samples in all four classes. The oversampling done by ADASYN increased to 2396, 2431, and 2405 samples in class 1, class 2, and class 3, respectively, while 2369 samples were in class 0. Though all three techniques generate synthetic samples to handle class imbalance, SMOTE and SMOTEN generate synthetic samples uniformly by ensuring each minority class has an exact number of samples as the majority class, leading to a perfectly balanced dataset. On the other hand, ADASYN works by adapting synthetic samples for minority classes that are closer to the classification decision. The grand‐averaged results obtained from oversampling techniques after feature optimization are listed in Table 4. The performance of machine learning models was reduced in the case of SMOTE and ADASYN, as they might have introduced unrealistic synthetic samples, while SMOTEN preserved important patterns in the dataset when generating synthetic samples for minority classes. SMOTEN outperformed in improving the accuracy of machine learning classifiers by computing a train accuracy of 94.16% and test accuracy of 92.07% for k‐Nearest Neighbors. The machine learning models of the Decision Tree and Random Forest performed comparably to kNN, with the Decision Tree having a train accuracy of 90.65% and test accuracy of 90.97%, while the Random Forest had a train accuracy of 91.57% and test accuracy of 91.46%. The difference in test and train accuracy was observed to have a very small gap, ensuring the models did not overfit during the training process. In addition to training and test accuracy, grand‐averaged precision, recall, and F1‐score were also evaluated to examine how well the model detects bruxism/teeth grinding by avoiding the false prediction, because accuracy alone can be misleading as it does not consider class distribution when evaluating model performance. The precision, recall, and F1‐score for k‐Nearest Neighbor with Linear Discriminant Analysis and SMOTEN were found to be 92.71%, 92.07%, and 92.21%, respectively. This ensured the model generalized results in the classification of bruxism among other masticatory muscle activities.

**TABLE 4:** The train accuracy, test accuracy, precision, recall, and f1‐score (in percentages) of different machine learning classifiers after applying oversampling techniques of SMOTE, SMOTEN, and ADASYN.

Resampling method	Model	Train accuracy	Test accuracy	Precision	Recall	F1 score
SMOTE	kNN	89.32	84.83	85.15	84.83	84.26
	Logistic Regression	38.87	38.72	38.43	38.72	37.63
	Naive Bayes	37.28	37.43	41.94	37.43	37.39
	Decision Tree	43.93	43.71	43.49	43.71	43.54
	Random Forest	63.27	60.54	61.37	60.54	60.62
SMOTEN	kNN	94.16	92.07	92.71	92.07	92.21
	Logistic Regression	84.02	83.92	84.00	83.92	83.57
	Naive Bayes	82.87	82.79	83.05	82.79	82.33
	Decision Tree	90.65	90.97	92.62	90.97	91.26
	Random Forest	91.57	91.46	93.37	91.46	91.78
ADASYN	kNN	88.37	83.66	84.49	83.66	82.96
	Logistic Regression	37.21	37.55	37.17	37.55	36.51
	Naive Bayes	34.59	34.39	37.51	34.39	33.77
	Decision Tree	41.37	41.71	42.06	41.71	41.79
	Random Forest	62.37	58.96	59.14	58.96	58.68

Since k‐nearest Neighbors, Decision Tree, and Random Forest gave comparable results, these three machine learning models were further evaluated with their confusion matrices and k‐fold accuracies over each of the 10 folds, as shown in Figure 12. Since our goal is to minimize the misclassification and maximize the correct prediction, the diagonal values of the confusion matrix, representing true positives, should be high, and off‐diagonal values should be comparatively very low. As presented in Figure 12, kNN, DT, and RF showed a high number of correct predictions by representing high true positive values for class 1 (Bruxism class) and true negative values for classes 0, 2, and 3, i.e., rest, talking, and chewing, respectively. Along with confusion matrices, k‐fold accuracy curves were also plotted to evaluate the stability, consistency, and generalization of machine learning models across different folds. The plot of 10‐fold accuracy illustrates how accuracy varies across different folds, indicating how well the model generalizes to unseen data. It was observed that initially, the models showed low accuracy up to 3 folds. The sharp rise is likely due to limited data in early folds or an imbalanced class distribution. After 3 folds, the accuracy remained consistently high (~0.95) for the remaining folds. This indicates good generalization and low variance, meaning the model performs well on different test sets. Hence, the 10‐fold cross‐validation plot for all three classifiers, kNN, DT, and RF, showed that the classifier learns quickly and maintains high and stable accuracy, leading to the generalization of our machine learning models in classifying teeth grinding activity as bruxism across different folds.

Lastly, the subject‐wise accuracy was computed only using the best‐performing machine learning models, by employing 10‐fold cross‐validation and optimizing features through LDA. The results for kNN are shown in Figure 13 for three trials of all 10 subjects, with an average accuracy of 84.33%. The inter‐subject accuracy comparisons were not computed at each step of selecting features and oversampling techniques to prevent redundant or potentially misleading interpretations from suboptimal configurations. The maximum accuracy observed was 91.94% and the minimum accuracy was 74.72%. Since the bruxism‐like activity was simulated, variation exists in how participants followed the clenching instructions (in terms of intensity, duration, or timing), which could affect data consistency and, in turn, the classification accuracy. The inter‐subject accuracy range likely reflects a combination of individual variability in hemodynamic responses recorded using fNIRS and related inconsistencies in optode‐skin contact and differences in task compliance. The encouraging performance metrics (grand‐averaged and individual subjects) support the feasibility of fNIRS in classifying jaw activities of talking, chewing, and simulated bruxism characterized by forceful jaw clenching and teeth grinding, but the generalizability and clinical applicability of these findings are constrained by several methodological limitations.

### 4.1 Limitations

This study was done as an exploratory preliminary study to evaluate the feasibility of fNIRS‐based monitoring of bruxism. But the experiment was recorded in a controlled setup on healthy participants simulating bruxism‐like jaw movements rather than actual patients exhibiting clinical bruxism. Though the subjects were trained to perform bruxism‐like activity, there is still a lack of the involuntary, rhythmic patterns that are often associated with sleep‐related neural and muscular characteristics of true bruxism. Each participant completed three trials, which is a modest number for capturing the variability of neural responses and jaw movements, but larger datasets with more repetitions per condition and subject would enhance robustness and generalizability of the findings to clinical populations. Even though the dataset to train machine learning classifiers is large enough to avoid overfitting, still this preliminary study comprises a dataset from a small sample size of 10 subjects. This causes the risk of interpretation bias, which limits the statistical power and generalizability of the findings. The study focused on bruxism‐like jaw movement only and aimed to distinguish how these jaw movements differ from the daily activities of the mandible, such as talking and chewing. The study does not contribute to diagnosing and capturing the full complexity of the neurophysiological underpinnings of bruxism and does not explicitly distinguish between sleep bruxism (SB) and awake bruxism (AB), which have distinct etiologies, pathophysiology, and diagnostic frameworks; rather, only motor activity patterns were studied. While EMG signals were recorded alongside fNIRS to support motor task detection, gold‐standard diagnostic tools such as polysomnography (PSG) or EMG‐based RMMA scoring were not used to confirm bruxism activity. Furthermore, as data collection occurred only in short daytime sessions, the system's capability for overnight monitoring or longitudinal application remains unexplored. This study offers promising evidence for the feasibility of fNIRS in detecting bruxism‐like jaw motor activity; its findings should be interpreted while considering the methodological limitations outlined above. Future work involving clinical bruxers, gold‐standard validation methods, and larger, more diverse samples is essential for advancing toward practical diagnostic applications of sleep and awake bruxism.

## 5 Conclusion

In this research, the potential of functional Near InfraRed Spectroscopy (fNIRS) is assessed in monitoring and detection of rhythmic bruxism‐like masticatory muscle activities and other masticatory activities of chewing and talking. The cortical activity in the motor cortex region was recorded, and corresponding hemodynamic states were assessed through brain activation maps across activities of rest, talking, chewing, and simulated‐bruxism‐like teeth grinding and jaw clenching. These activities were then classified using machine learning classifiers, namely, k‐nearest Neighbors, Logistic Regression, Naive Bayes, Decision Tree, and Random Forest. The performance of machine learning models was compared by optimizing the feature set of fNIRS data and augmenting the imbalance in classes of rest, talking, chewing, and grinding using oversampling techniques. The k‐nearest Neighbors, Decision Tree, and Random Forest outperformed and gave comparable results with a grand‐averaged test accuracy of 94.16%, 90.65%, and 91.57% for k‐NN, DT, and RF, respectively, when Linear Discriminant Analysis and SMOTEN were applied. The intersubject accuracies were compared, which were in the range of 91.94% and 74.72%. These findings suggest that fNIRS can serve as a reliable clinical tool in simulated‐bruxism‐related RMMA assessment and detection among other activities associated with masticatory muscles. However, the results from this preliminary study should be interpreted with caution, considering the limitations of a small sample size and simulated bruxism in healthy individuals instead of patients, which may not exactly replicate the responses from bruxers. The incorporation of clinical populations from diverse age groups can be explored for generalization and clinical relevance to the condition of bruxism.

## Author Contributions

Noor Fatima contributed to the study design, data collection, analysis, and manuscript writing. Zia Mohy Ud Din provided technical expertise, guided the methodology, and reviewed the manuscript. Abdullah Al Aishan assisted with data analysis and manuscript drafting. Jahan Zeb Gul contributed the initial idea, research direction, and final draft review.

## Ethics Statement

The study was approved by the Department of Mechatronics and Biomedical Engineering, Air University, Islamabad, Pakistan, with ethical approval number AU/EA/2024/32/343.

## Conflicts of Interest

The authors declare no conflicts of interest.

## Data Availability

Due to ethical considerations, the data are available only upon special request to the authors.
